# HIV associated thrombocytopenia, misdiagnosed as thrombotic thrombocytopenic purpura: a case report

**DOI:** 10.1186/1757-1626-2-175

**Published:** 2009-10-29

**Authors:** Farzin Khorvash, Alireza Emami Naeini, Mohadesseh Behjati, Mohammad Jalali

**Affiliations:** 1 Department of Infectious and Tropical Diseases, Isfahan University of Medical Sciences, Isfahan, Iran; 2 Resident of Cardiology, Isfahan University of Medical Sciences, Isfahan, Iran; 3 School of Health, Isfahan University of Medical Sciences, Isfahan, Iran

## Abstract

**Introduction:**

Thrombocytopenia is one of the protean hematological manifestations of infection by human immunodeficiency virus. We present a case where diagnosis of human immunodeficiency virus infection was delayed because thrombocytopenia was the only presenting feature.

**Case presentation:**

A female presented with chief complaint of fever, productive coughs weight loss and nocturnal sweating; was detected to have thrombocytopenia on a complete blood count, performed five months ago. Her other clinical chemistry and hematological investigations were normal. She was previously diagnosed as having TTP, and had received 55 sessions of plasma exchange before presenting to us. At this time she was detected to be HIV positive, had CD4+ counts of 26/mm3, treated with antiretroviral drugs and Cotrimoxazol, and discharged asymptomatically.

**Conclusion:**

HIV should be suspected in all cases of unexplained thrombocytopenia.

## Introduction

Hematological manifestations of human immunodeficiency virus (HIV) infection have a broad spectrum, ranging from mild disturbance in quality of life to life-threatening conditions [1]. Here, we present a case with thrombocytopenia where diagnosis of HIV infection was missed on initial evaluation.

## Case presentation

A 29-year-old Iranian female, presented to infectious disease services with chief complaints of recurrent fever since past five months and productive cough, of 20 days duration. Five months ago, while being evaluated for fever her complete blood count (CBC) had revealed thrombocytopenia. For evaluation of thrombocytopenia, a bone marrow examination was done which showed erythroid hyperplasia. A provisional diagnosis of thrombotic thrombocytopenic purpura was made, and the patient underwent 55 sessions of plasma exchange, over next two months. Her fever had subsided by this time. Three months later (five months after onset of her initial symptoms), she presented with fever, nocturnal sweats, productive cough, weight loss, weakness and generalized aches and pains, productive coughs. Her body temperature was 39°C, Pulse rate: 108, Blood Pressure: 110/70 mmHg. On physical examination, she had pale sclera, oral thrush, neck stiffness, crackles on lung fields, hepatosplenomegaly and tender right upper quadrant of abdomen. A repeat CBC showed pancytopenia (WBC: 2400/μl with 66% PMN, 34% Lymph, Hb:5.6 g/dl, PLT:64800/μl), ErythrocyteSedimentationRatewas68.BUN/Cr, haptoglubolin and billirubin (total, direct and indirect) were in the normal range.

Coomb's test was also negative. On reviewing the history again we found that four years ago patient's sexual partner was an intravenous drug user. This prompted us to do an HIV test in her, which was found to be positive. Her CD4 counts were low (26/mm3). Subsequently we performed a bone marrow biopsy and bone marrow aspiration, which were normal. Her cerebral spinal fluid analysis was normal with negative smear and culture. Abdominal ultrasonography showed hepatosplenomegaly and presence of a renal stone. Bilateral para-hilar infiltrations were seen on chest X-ray. Broncho-Alveolar Lavage smear was positive for Pneumocystis Jirovecii antigen. Antiretroviral drugs (Zidovudine, Lamivudine and Nelfinavir) were started in combination with Trimethoprim-Sulfamethoxazole for the patient. Her fever subsided and was discharged with a diagnosis of HIV associated thrombocytopenia. On follow up her platelet counts improved (WBC: 3500/μl) with 62% PMN, 38% Lymph, Hb: 9.0 g/dl, PLT: 105000/μl, CD4: 102/mm3) and lesions on her chest x-ray resolved.

## Discussion

Increasing the complexity of HIV infection, different hematological manifestations can be seen [1], in which HIV-related thrombocytopenia (Tr-HIV) is one of them [2], often asymptomatic [3], but may be associated with a variety of bleeding abnormalities [4] and with a high morbidity [2]. Despite of the common prevalence of HIV- induced thrombocytopenia [5], profound thrombocytopenia is totally rare (1.5%) [6]. This problem is truly a medical challenge in vulnerable population, especially by the limited therapeutic options and the absence of intervention protocol for HIV subjects [7]. It's not age and sex dependent and interestingly [2], in contrast with anemia and neutropenia [9], even it's not dependent to the stage of infection [2]. Different mechanisms were described for this, but the two most common ones are rapid destruction by circulating immune complexes (especially profound ones) [3], and insufficient production (especially in mild ones) [2]. But, some other rare causes are also described as TTP [9]. Although knowing the exact mechanism in each patient seems difficult, diagnosis of the mechanisms and causes of the thrombocytopenia seems necessary for the optimal management of the patient [8]. For example in the cases of immune-mediated thrombocytopenia a combination of zidovudine, corticosteroids, IVIG, splenectomy and some times platelet transfusion may be helpful [8]. TPO, although investigational, is useful for the cases of decreased platelet production [10], and plasmaphresis for the cases conflicted by TTP, are used effectively [11]. Some other modalities, such as interferon, vincristine, danazol, low-dose splenic irradiation and staphylococcal protein A and immunoabsorption have been used with limited success [12]. But it's well understood that HIV-related thrombocytopenia (HIV-TP) responds to antiretroviral therapy [13] and most studied have shown that zidovudine (AZT) increased platelet production, especially in combination therapy (ART) [14].

By the presence of great mimickers, the real face of HIV induced thrombocytopenia may be masked in different situations, as our case, in which the diagnosis of TTP was made for her. HIV is one of the causes of TTP, especially in the late stages [10], and plasmaphresis is also a good therapeutic option [11]. Ignoring the complications of misdiagnosis for the patient, the role of plasmaphresis facilities in AIDS epidemic, especially in developing countries [15], is not negligible.

It is not clear why the first clinician diagnosed TTP, not ITP for the patient, only based on the fever and thrombocytopenia. And our message is the value of considering HIV as a cause of thrombocytopenia seems a matter needs more attention than paid previously, especially in developing countries. As a suggestion, taking an HIV test may increase the plasma exchange safety for healthy persons undergoing.

## Conclusion

Hence HIV positive cases are increasing, especially in the developing countries; consequently, the great clinical suspicion is needed to consider it as a cause of thrombocytopenia. The importance of this matter is not only from diagnostic and therapeutic aspects, but also ethical concerns behind; non HIV cases who undergo plasma exchange. So, the significant role of proper history taking will become elucidate once again.

## Abbreviations

CBC: Cell Blood Count; TTP: Thrombotic Thrombocytopenic Purpura; ITP: Immune Thrombocytopenic Purpura; HIV: Human Immunodeficiency Virus; AIDS: Acquired Immunodeficiency Syndrome; ART: Active Retroviral Therapy; Tr-HIV: Thrombocytopenia related HIV; CXR: Chest X Ray; IVIG: Intra Venus Immunoglobulin; PLT: Platelet.

## Consent

Written informed consent was obtained from the patient for publication of this case report and accompanying images. A copy of the written consent is available for review by the Editor-in-Chief of this journal.

## Competing interests

The authors declare that they have no competing interests.

## Authors' contributions

FK is corresponding author of the manuscript and the first case manager, AEN helped in case management, MB and MJ helped in righting of article and case management.
